# Audit Cycle on Medical Reviews of Seclusion in Medium and Low Secure Learning Disability Units

**DOI:** 10.1192/bjo.2022.435

**Published:** 2022-06-20

**Authors:** Sruthi Easwaran Iyer, Abigail Williamson

**Affiliations:** Mersey Care NHS Foundation Trust, Liverpool, United Kingdom

## Abstract

**Aims:**

Seclusion is defined as “the supervised confinement and isolation of a patient, away from other patients, in an area from which the patient is prevented from leaving, where it is of immediate necessity for the purpose of the containment of severe behavioural disturbance which is likely to cause harm to others”. Patients in seclusion require reviews at the frequency set out in the Mersey Care NHS Foundation Trust policy, “The use of seclusion and long-term segregation” (SD28). This is based on the requirements set out in the Chapter 26 of the Mental Health Act 1983 Code of Practice (2015).This audit will look at whether medical reviews for secluded patients in the secure learning disability wards meet with the expectations set out in the Trust Policy. In doing so, the audit will establish whether medical reviews of seclusion meet and uphold the guiding principles of the Mental Health Act Code of Practice as highlighted in Chapter 26.110.

**Methods:**

Retrospective audit that collected data from inpatients on secure learning disability wards in Mersey Care. After reviewing data, we actioned plans which involved educating colleagues working in secure services. This was re audited after three months. One month of seclusion reviews was audited in each cycle, which equated to 39 reviews in the first cycle and 100 reviews in the second.

**Results:**

The re-audit data showed an improvement in most parameters.

Re-audit showed that 66% (34%) of the seclusion reviews had an initial medical review within the first hour. The on call consultant was informed in 60% (50%) of the situations and 4 hourly reviews took place in 66% (50%) of scenarios. All MDT reviews took place within 24 hours, Responsible Clinician was present in 100% (67%) of reviews.

34% (33%) of MDT reviews had only 2 MDT members.

There was 100% compliance with reviewing physical health in both audits. 100% (90%) of the reviews commented on mental health, 72% (20%) commented on medications used, 51% (39%) of reviews commented on level of observations and 89% (48%) included risk assessment. 95% (92%) of reviews assessed need for continuing seclusion. 84% (59%) of reviews commented on reducing restriction in seclusion.

**Conclusion:**

This audit cycle has focused on the quality of medical reviews and not just the frequency. The improvement in practice will strengthen the safeguard provided by these reviews.

